# Development of a portable testing chamber to assess imaging performance of laparoscopes in low- and middle-income countries

**DOI:** 10.1117/1.JBO.30.1.016001

**Published:** 2025-01-30

**Authors:** Anne Christine Barnes, Michele Kaluzienski, Tri Quang, Jason Chen, Surabhi Singh, Wilhelm Smith, Talya Simcox, Paula Kworekwa, Rebecca Kaaya Nansubuga, Robert Ssekitoleko, Tamara N. Fitzgerald, Jenna L. Mueller

**Affiliations:** a University of Maryland, Department of Bioengineering, College Park, Maryland, United States; b Makerere University, Biomedical Engineering Unit, Department of Physiology, School of Biomedical Sciences, College of Health Sciences, Kampala, Uganda; c Duke University School of Medicine, Department of Surgery, Durham, North Carolina, United States; d University of Maryland School of Medicine, Marlene and Stewart Greenebaum Cancer Center, Baltimore, Maryland, United States

**Keywords:** laparoscopic surgery, biomedical optics, low- and middle-income countries, global surgery, frugal medical device, global engineering

## Abstract

**Significance:**

Laparoscopic surgery is generally unavailable in low- and middle-income countries (LMICs) due to the high cost of installation and lack of qualified personnel to maintain and repair equipment. We developed a low-cost, durable, reusable laparoscopic system, called the KeyScope laparoscope, for use in LMICs. To reliably build and service the KeyScope in LMICs, a portable testing chamber (PTC) is needed to assess image performance.

**Aim:**

A PTC was developed to characterize KeyScope laparoscope performance in LMICs.

**Approach:**

Images of standard resolution, color accuracy, distortion, and depth of field (DOF) targets were captured in both a standard optical bench setup (OBS) and the PTC. Measurements from the OBS and PTC were quantified and compared using standard software (ImageJ and Imatest). To further reduce cost, alternative paper imaging targets were identified and compared with standard glass targets. To improve usability, MATLAB applications (apps) were developed to automate image analysis and reduce cost.

**Results:**

The PTC achieved similar results compared to the OBS for the image quality metrics, distortion and DOF. Further, the PTC presented similar results to the OBS for resolution at 4 to 7 cm working distances and improved resolution at periphery working distances of 3 and 10 cm. Color accuracy values were also improved in the PTC compared with those measured in the OBS. The low-cost resolution, color accuracy, and distortion targets resulted in similar image quality results to the standard image quality target. MATLAB apps produced similar results to Imatest and ImageJ software and decreased the time to complete image quality test analysis.

**Conclusion:**

The low-cost portable design of the PTC will facilitate the translation of the KeyScope by enabling accurate and fast characterization of laparoscopic imaging performance in LMICs.

## 1 Introduction

Laparoscopic surgery is widely used for most procedures within the chest and abdominal cavity.^1^ Standard laparoscopic systems include a high-resolution camera and a light source coupled to a fiber optic probe, allowing fine surgical instruments to be inserted via keyhole incisions, eliminating the need for open surgical approaches.^2^ Laparoscopic surgery is the standard of care in high-income countries (HICs) due to its well-documented benefits, including reduced bleeding, shorter patient recovery time, and decreased surgical site infection,^3^ all of which improve surgical outcomes and reduce healthcare system costs.

Although laparoscopic surgery is widely used in HICs, low- and middle-income countries (LMICs) lack access. Laparoscopy is needed in LMICs, particularly because surgical site infection rates are five times higher^4^ and long hospitalization can be financially damaging to families.^5^^–^^7^ These difficulties could be mitigated by the implementation of laparoscopy, but several barriers remain, including the high cost of initial purchase, supplies, and local expertise needed for system maintenance, and the lack of laparoscope design features tailored to LMIC resources.^5^^,^^8^ The current laparoscopic setup can cost upward of $130,000 for the laparoscope, viewing monitors, and corresponding equipment, preventing purchase in LMIC healthcare systems.^9^^,^^10^ In addition, the standard of care laparoscopes lack design considerations for LMIC resource compatibility. For example, they require continuous power, posing issues when used in countries that experience power outages,^11^ as well as incompatibility with submersion sterilizations, which is the primary sterilization method in LMICs.^12^

We previously developed a low-cost laparoscope called the KeyScope, created specifically for LMIC environments.^13^ The KeyScope utilizes light-emitting diodes (LEDs) and a complementary metal-oxide-semiconductor sensor (CMOS) detector placed at the tip of the laparoscope, eliminating the need for fragile fiber optic cables and lens systems. The KeyScope is powered via connection to a laptop computer with a universal serial bus (USB) cord, and the image is displayed on a laptop computer, removing the need for constant power and expensive monitors. The KeyScope is waterproof, so it can be sterilized through submersion techniques, as is common in LMICs.^14^ The current KeyScope design has been derived through an iterative human-centered design process with surgeons^15^; this included surveying LMIC surgeons to determine clinical needs^16^ and modifying device specifications and performance following feedback from surgeons who used the KeyScope in porcine models.^17^ Our team is working with a medical device company in Uganda and partnering with local distributors to facilitate local capacity building in medical device design and manufacturing. Because the manufacturing and quality assurance testing of the KeyScope will be performed in Uganda, where OBSs are not readily available, a reliable and consistent testing chamber is needed to evaluate the performance of each manufactured KeyScope prior to clinical use.

Various image quality parameters are necessary to characterize a laparoscope’s performance, including resolution, image distortion, color accuracy, and depth of field (DOF). Previously, these image quality parameters were quantified in a custom optical bench setup (OBS) in which collimated white LEDs with diffusers were set between 20 deg and 40 deg (relative to the device being tested) to provide uniform lighting and minimize specular reflection from the imaging target (per ISO 12233:2017 guidelines).^13^ The OBS used a large, immovable optical table and multiple optical and opto-mechanical parts, which are cost-prohibitive for LMIC implementation. In addition, the OBS was not enclosed and was subject to variations in ambient lighting. The cost, immobility, and variability of an OBS hinder the overall goal of the KeyScope.

To enable characterization of the KeyScope in LMICs, we describe the design of a low-cost, portable testing chamber (PTC). The overall size, lighting conditions, and materials were carefully selected to ensure that the PTC yields comparable results to the OBS while also meeting three criteria: low weight (<10  lbs), low cost (<$1000), and flexibility. Flexibility was defined as a design that enabled all image quality tests to be conducted with minimal modification or switching out of components. Foam walls with a black exterior were used to block ambient light, and a white interior was chosen to enable uniform illumination of optical targets. Overhangs and interlocking joints were added between walls to ensure that testing conditions remained constant regardless of external lighting conditions. The position of the LEDs was systematically tested to ensure their placement yielded consistent and even light exposure for all target positions within the chamber. Equivalency was then tested between the PTC and the OBS. Costs were decreased by replacing glass targets with paper or plastic targets, and demonstrating equivalence. Previously, the image quality targets were analyzed through a time-consuming manual method (in ImageJ) or through expensive image quality analysis software (Imatest). To decrease the time and cost of image quality testing analysis, MATLAB apps were developed and validated to enable rapid, semi-automated analysis of various imaging targets.

## 2 Methods

### 2.1 Portable Testing Chamber Design

**Fig. 1:**
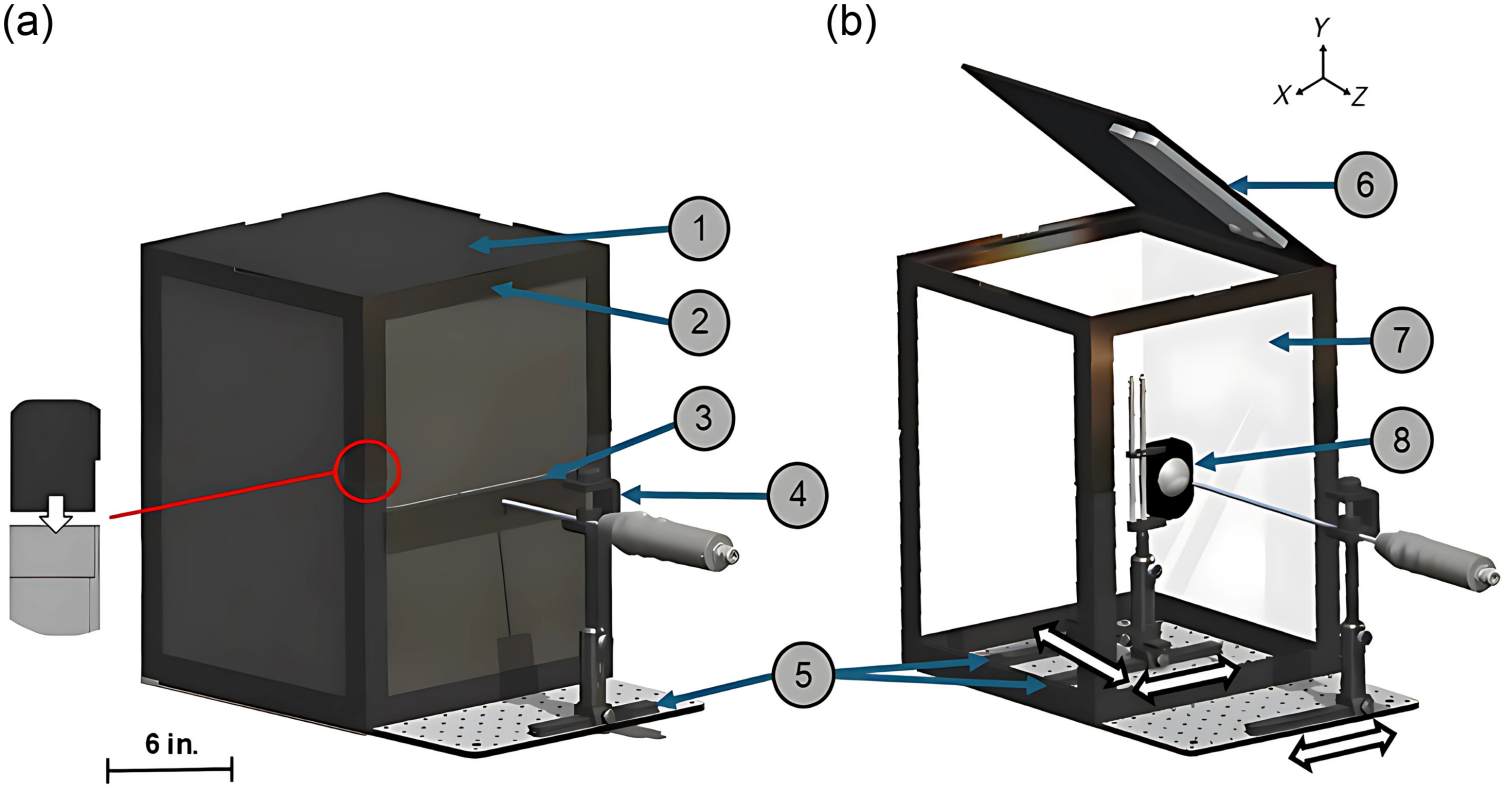
Diagram of the portable testing chamber (PTC). (a) Closed configuration of the PTC. The components included (1) black cardstock exterior wall, (2) 3D-printed interlocking frames, (3) a brush slit through which the KeyScope is inserted, (4) KeyScope holder attached to a rail slider, and (5) sliding rails. The red circle highlights an interlocking joint for the 3D-printed frame to reduce light penetration. (b) Open configuration of the PTC. The components included (6) two LED arrays, (7) a white foam interior wall, and (8) a target holder affixed to sliding rails. White arrows indicate the possible direction of movement on the sliding rails.

The overall design of our PTC, as seen in Fig. 1, includes a rectangular enclosure consisting of 16×12  in. white foam boards coated with black cardstock and joined by 3D-printed interlocking frames. The white foam boards were chosen to increase light scattering within the PTC and achieve uniform illumination of the optical targets.^18^ A thick layer of black cardstock was adhered to the outward-facing side of the boards, and interlocking frames were added to eliminate ambient light leakage inside the PTC and ensure that the testing conditions remained consistent. The base was a 12×24  in. aluminum optical breadboard plate (Newport Corporation, Irvine, United States) with three sliding rails where two 9-in. rails (Thorlabs, RLA1800, Newton, United States) were used for target holder movements in the x-direction, and one 6-in. sliding rail (Thorlabs, RLA0600) was affixed on top of the 9-in. rails to allow for z-direction movements. A rail slider post holder (Thorlabs, RC1) was used as the target holder base, and various optical targets were secured in place via another target holder (Thorlabs, TRA3). The laparoscope entered the PTC through a slit that was lined with a brush (Tambee, New York, United States) to reduce any ambient light penetration. Similar to the target, the laparoscope rested on a 6-in. rail for movement in the x-direction and was secured with a clamp mount (Novoflex, UNIKLEM-42, Kolkata, India). Finally, the targets in the PTC were illuminated via two LED arrays (Uizyfit, Shenzhen, China) magnetically attached to the top.

### 2.2 Uniform Illumination of Testing Targets

**Fig. 2:**
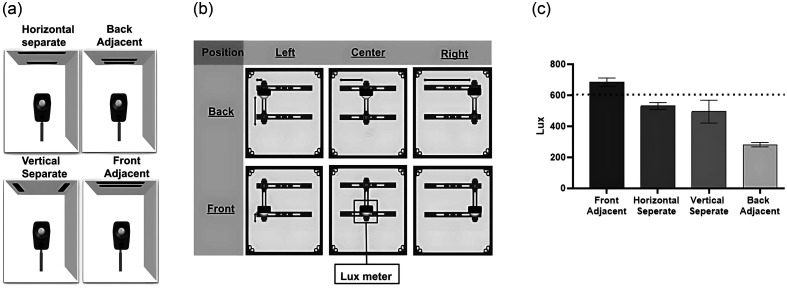
Methods to assess various LED array positions. (a) Different LED array positions on the ceiling of the PTC. LED positions included horizontal separate, vertical separate, back adjacent, and front adjacent. (b) Lux meter positions to measure illumination within the PTC. The lux meter was placed in the target holder, and the lux generated from each LED array configuration in panel (a) was measured at each target position in panel (b). Possible target positions included left-back, center-back, right-back, left-front, center-front, and right-front. (c) LED position assessment. The average lux values obtained across lux meter positions, as seen in panel (b), within the PTC for each LED array position. The front adjacent displayed the lux that best matched optical bench lux values, as indicated in the figure with a dotted line. Error bars indicate standard deviation for a sample size of n=6.

To determine the optimal lighting arrangement, various LED positions were tested throughout the PTC [Fig. 2(a)]. The objective was to find the position that provided the most uniform lighting and most closely matched the lux values measured in the OBS, which was ∼600  lux. For each LED position, the lux was measured from six target positions with a lux meter (Voltcraft, LX-1108) [Fig. 2(b)]. The front adjacent configuration provided the lux value closest to the OBS while simultaneously providing the most uniform lighting (as evidenced by the smallest standard deviation), which justified our choice to place the LEDs in the front adjacent position of the PTC [Fig. 2(c)].

### 2.3 Portable Testing Chamber Validation

To validate the design of the PTC, we compared its performance with our standard OBS. The standard OBS setup has been previously described in detail.^13^ Briefly, it contains an XY-axis translation stage, a Z-axis translation stage, collimated white LED sources placed at 20 deg to 40 deg relative to the KeyScope for uniform illumination (per ISO 12233:2017 guidelines), a v-mount clamp for holding the tested device, and a dovetail sliding rail for coarse x-axis adjustment. Images of various test targets were acquired with our latest generation KeyScope in both the OBS and PTC as described below.^15^

#### 2.3.1 Resolution

Images of a USAF 1951 resolution target (Thorlabs, R2L2S4P) were captured with our latest generation KeyScope in both the OBS and PTC at working distances ranging from 3 to 10 cm (the range of working distances commonly used during laparoscopy).^13^ The smallest discernible line width the camera can resolve was determined with the open-source software, ImageJ (University of Wisconsin – Madison), as illustrated in Fig. S1 in the Supplementary Material. A line was drawn through each element of a group, and the pixel values were plotted using the “plot profile” function in ImageJ. If the ratio of the peak (white pixels) to trough (black pixels) fell below 2:1, then that element number was considered the limit of resolution.

#### 2.3.2 Color accuracy

Color accuracy measurements in each setup were determined by imaging the RezChecker target (Imatest, CO) and quantifying the color accuracy using Imatest software (Imatest Master, 2022, Imatest). The difference between reference color space values and measured color space values from the images was calculated using the Euclidean distance equation. The resulting color error, $\Delta {E^{*}}_{\mathrm{ab}}$, accounted for luminance in the calculation, as seen in Eq. (1) :

$$\Delta E{*}_{ab}=[\sqrt{\left(\Delta L*{)}^{2}+(\Delta a*{)}^{2}+(\Delta b*\right)}],$$ (1)

where ΔL is the difference in luminance between the reference and measured data, and Δa* and Δb* are the color-opponent dimensions. $\Delta C{*}_{ab}$ color error, which does not account for luminance, was also calculated via Eq. (2)

$$\Delta C{*}_{ab}=[\sqrt{(\Delta a*{)}^{2}+(\Delta b*{)}^{2}}].$$ (2)

#### 2.3.3 Distortion

**Fig. 3:**
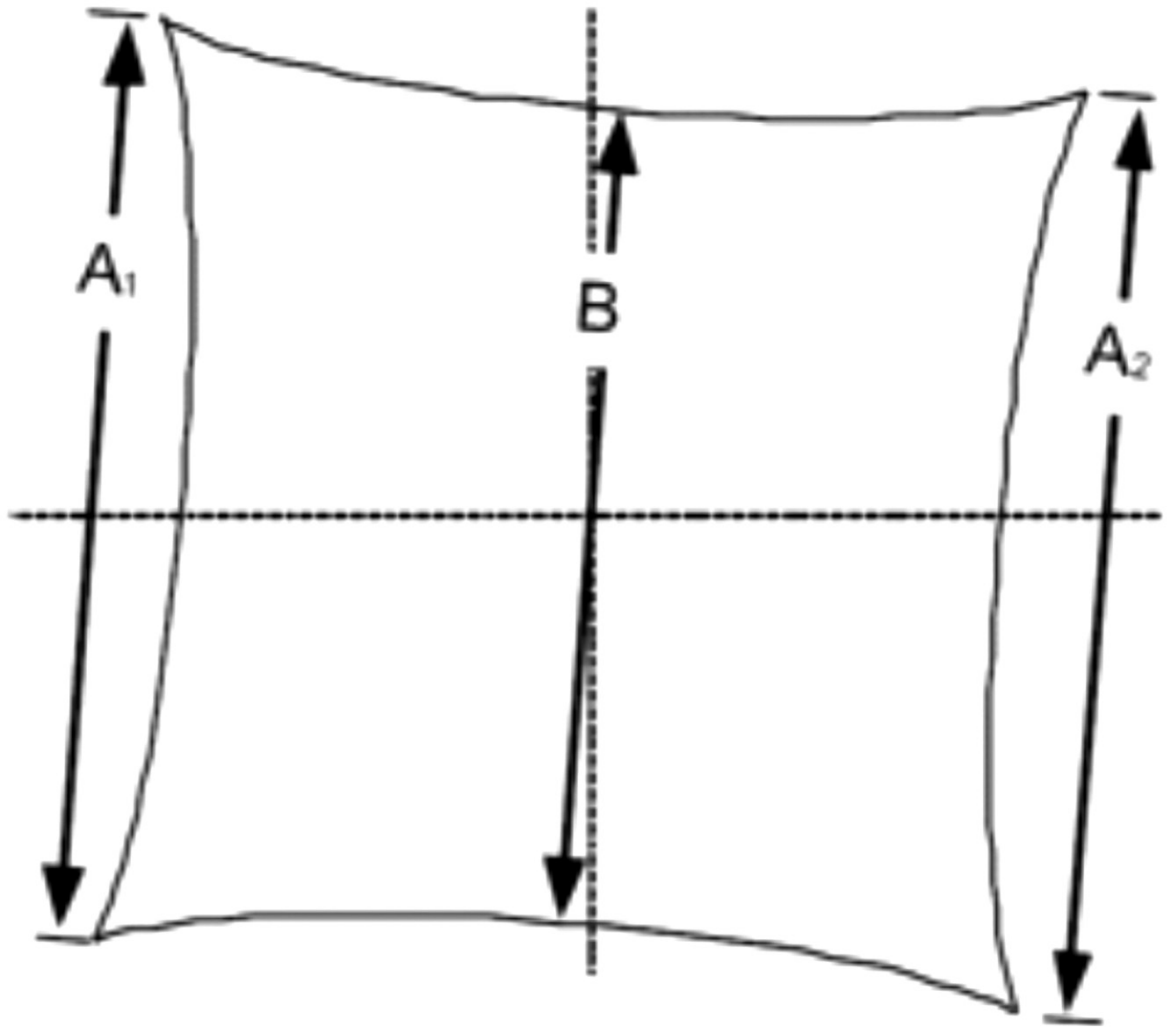
Mid-length, B, and side-lengths, $A_{1}$ and $A_{2}$, of a distorted image.

The radial lens distortion of the KeyScope was assessed by imaging the SFRplus geometric distortion target (Applied Image, QI-SFR15-P-CG) in the OBS and PTC. The images were analyzed using Imatest, which calculates the percentage of distortion using the standard mobile imaging architecture (SMIA) TV distortion equations

$$\%\text{Distortion}=\frac{100\times (A-B)}{B},$$ (3)

$$A=\frac{A_{2}-A_{1}}{2},$$ (4)

where $A_{1}$ and $A_{2}$ are the outer side lengths of a square, whereas B is the distance between the midpoints of the sides of the square. Equation (4) gives the average side lengths of the square (Fig. 3), whereas Eq. (3) gives the overall percentage of distortion.

#### 2.3.4 Depth of field

To complete the DOF analysis, we captured a series of images of the DOF target (Edmund Optics, 54-440, Barrington, United States) using the latest KeyScope at a working distance of 3 cm. Figure S2 in the Supplementary Material illustrates the manual calculation process. To manually calculate DOF, users upload the image to the ImageJ software and draw a vertical line along the horizontal lines on the right-hand side of the target, which has five-line pairs per millimeter. An intensity profile is generated using the “plot profile” function in ImageJ. The difference between the first full peak (white pixels) and trough (black pixels) is measured and accounted for as the initial value. The failure point is determined when the difference between a peak and subsequent trough falls below half of the initial value. The number of peaks between the start and the failure point is divided by 5 to obtain the DOF in millimeters.

### 2.4 Validation of Low-Cost Targets

**Fig. 4:**
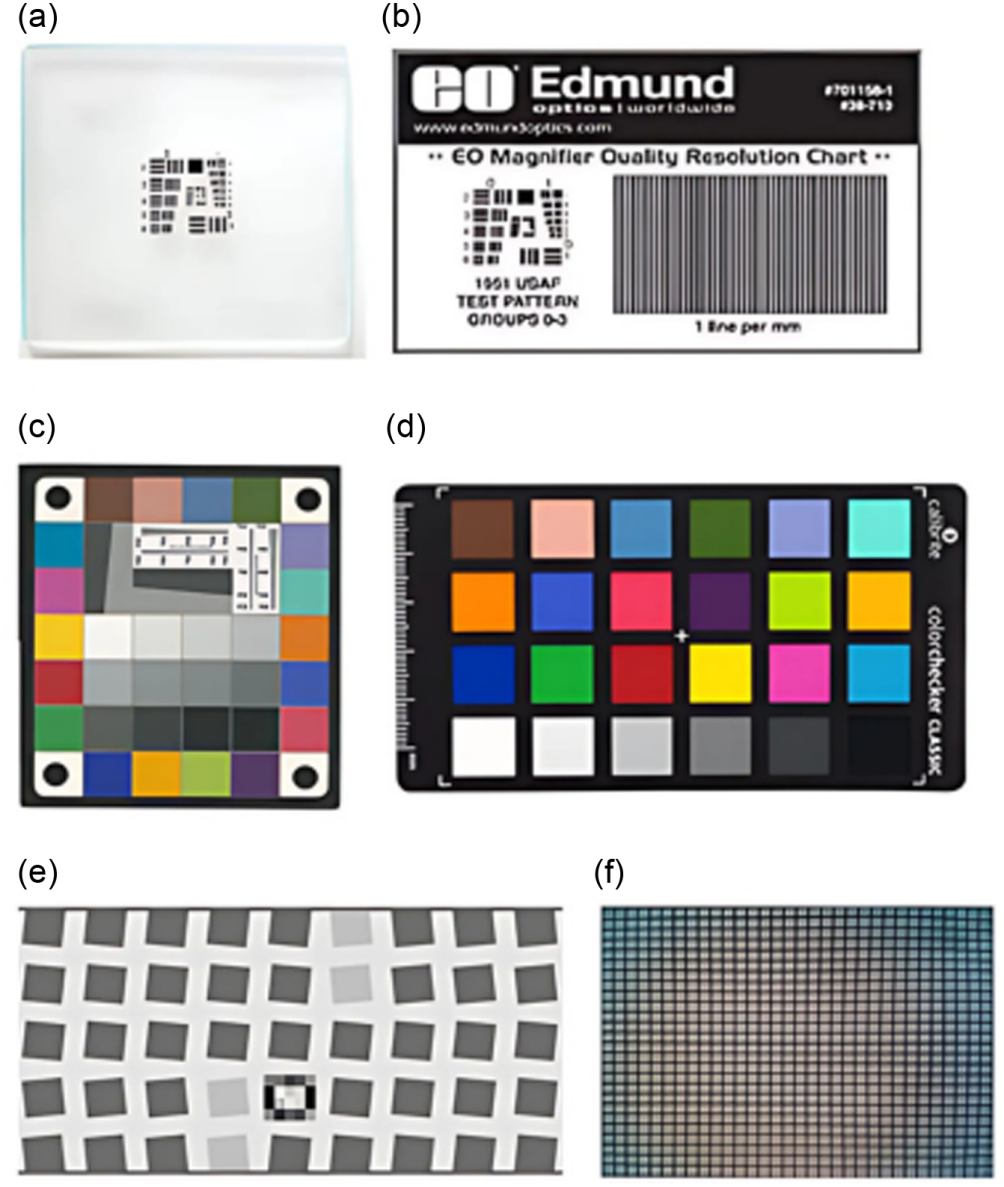
Glass versus paper image quality targets. (a) Glass USAF 1951 resolution target (Thorlabs, R2L2S4P). (b) 1951 USAF target pocket size (Edmund Optics, 38-710). (c) RezChecker target (Imatest, CO). (d) The ColorChecker Classic Mini (Calibrite, DE). (e) SFRplus geometric distortion target (Imatest, CO). (f) A 3D-printed grid target.

Because standard image quality test targets can be costly, more affordable paper or plastic alternatives were obtained and compared to glass targets. Each new paper/plastic target was imaged with the KeyScope in the PTC and compared to the results of the glass target. The paper resolution chart selected was the 1951 USAF target pocket size (Edmund Optics, 38-710). This target displays the same resolution line pairs as the glass target but is printed on paper, significantly reducing the cost from $200 to $9 [Figs. 4(a), 4(b)]. The ColorChecker Classic Mini (Calibrite, DE) was selected as our new color accuracy paper target due to its cardstock material, which reduced the price compared to the RezChecker target from $380 to $74 [Figs. 4(c), 4(d)]. For the distortion target, a printed grid previously developed by Wang et al.^19^ was used to replace the SFRplus distortion target [Figs. 4(e), 4(f)]. A 3D printing of the target greatly reduced the cost from $540 to <$1 (the cost of the 3D printing filament).

### 2.5 Development of Image Analysis Code

To reduce both the time spent on manual image analysis with ImageJ and money spent on image analysis with Imatest, we developed semi-automated MATLAB applications (apps) for resolution, DOF, color accuracy, and distortion measurements. MATLAB was selected due to the ease of app development, team familiarity, and access, which enabled efficient training and dissemination of apps to international collaborators. To validate our MATLAB apps, results were compared with the previous results obtained with ImageJ or Imatest. Specifically, five different users analyzed the same set of target images using both the ImageJ or Imatest method and the semi-automated MATLAB apps. The times to complete each analysis were also recorded. A more detailed explanation of the calculations and code is included in the Supplementary Material.

#### 2.5.1 Resolution analysis code

**Fig. 5:**
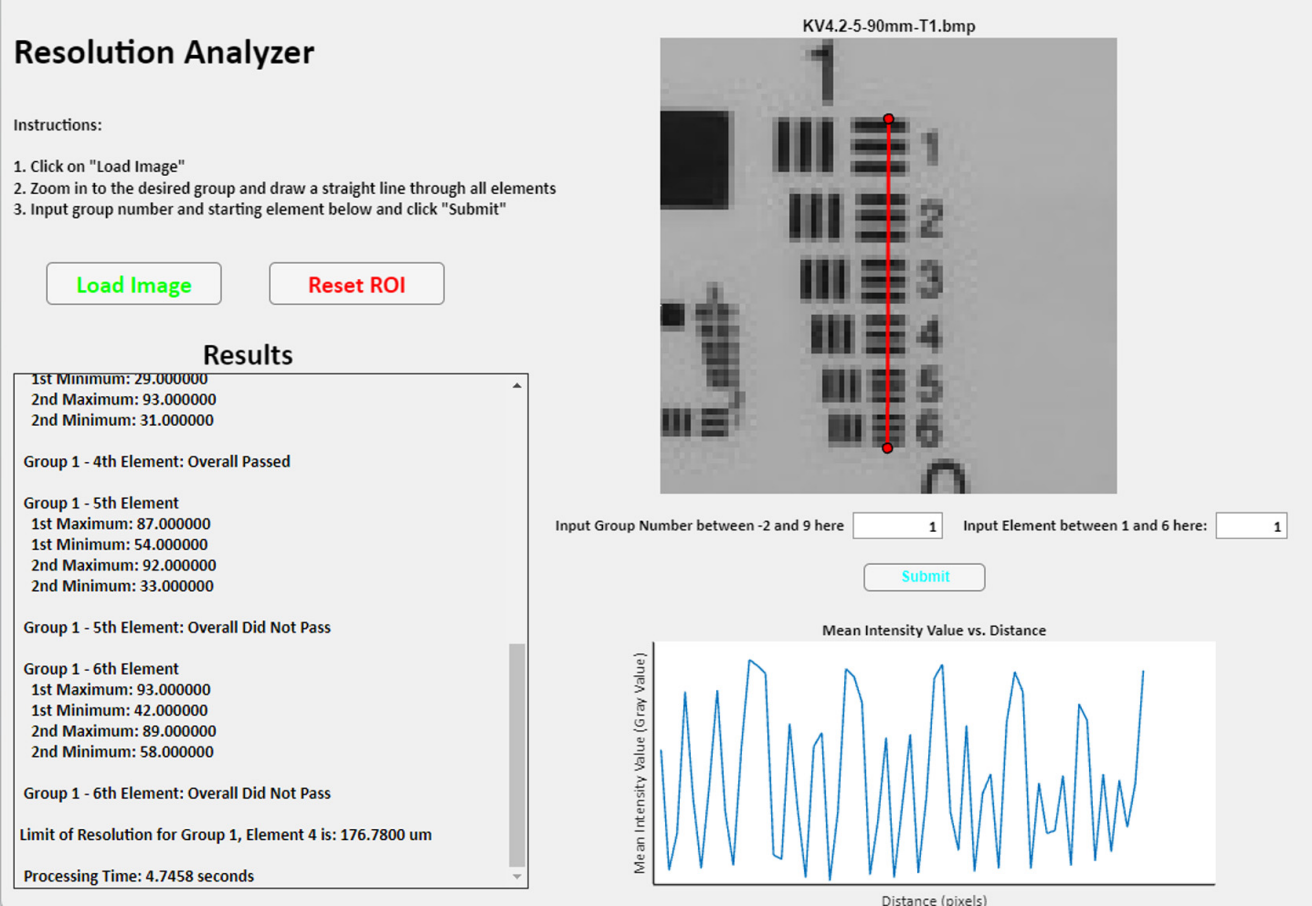
Semi-automated MATLAB Resolution Analyzer App user interface.

As described in the resolution methods above, ImageJ was used to analyze resolution target images. To increase the efficiency of this analysis, we designed a MATLAB code to automatically replicate these steps and provide the user with the lowest passing group and element and the limit of resolution in microns. The code follows the calculations seen in Fig. S1 in the Supplementary Material. To utilize the code, a user draws a line through an entire group on the resolution chart; the code will then tell the user which elements passed or failed the resolution analysis and provide the overall limit of resolution as seen in Fig. 5. It does so by plotting an intensity profile graph, identifying the intensity values of the peaks and troughs of each resolution line pair element, and then calculating if the element’s minimum intensity value is less than twice the value of the highest peak. The code identifies the smallest resolvable element and displays it to the user.

#### 2.5.2 Depth of field analysis code

The DOF image analysis process in ImageJ was replicated in our MATLAB app to increase analysis efficiency. Our semi-automated app (displayed in Fig. S3 in the Supplementary Material) requires the user to draw the line on the DOF target image, with the algorithm performing all subsequent calculations (described in Fig. S2 in the Supplementary Material). Once the user draws the line, an intensity plot is generated, similar to the “plot profile” function in ImageJ. The code calculates the range difference between the first peak and first trough, divides it by half, and assigns it as the range check variable. The code then iterates through all subsequent peak and trough ranges until the difference between the peak and adjacent trough is less than half of the range check. Once that failure point is found, the algorithm ensures that the following twenty iterations are also below the official range check to ensure a downward trend is followed; this eliminates any possible anomalies. Once that failure point is validated, the algorithm counts the number of peaks and divides by 5 to get the DOF in millimeters and displays this value. The semi-automated MATLAB app eliminates the need for users to do steps 3 to 5 in Fig. S2 in the Supplementary Material by hand.

#### 2.5.3 Color accuracy analysis code

Our MATLAB app for color analysis, shown in Fig. S4 in the Supplementary Material, calculates the color accuracy in the same manner as Imatest. Specifically, it determines the absolute color differences by calculating the absolute color difference [Eq. (1)] and absolute chroma difference [Eq. (2)]. In addition to calculating the absolute color difference and absolute chroma difference, the app, similar to Imatest, shows the difference between the reference color and the input color from the image. This allows the users to visualize the color difference.

#### 2.5.4 Distortion analysis code

Our semi-automated distortion MATLAB app, seen in Fig. S5 in the Supplementary Material, calculates the distortion similarly to Imatest through the SMIA TV distortion Eq. (2). The user selects the left-most, right-most, and middle points of the grid target image on the top and bottom grid lines. The code calculates $A_{1}$, B, and $A_{2}$ by finding the number of pixels between the top left endpoint and bottom left endpoint ($A_{1}$), the top middle endpoint and bottom middle endpoint (B), and the top right endpoint and bottom right endpoint ($A_{2}$). The algorithm averages the distance of $A_{1}$ and $A_{2}$ to get A. Finally, the code inputs variables A and B into the SMIA TV distortion Eq. (2) and displays this value to the user.

## 3 Results

### 3.1 Portable Testing Chamber Validation

**Fig. 6:**
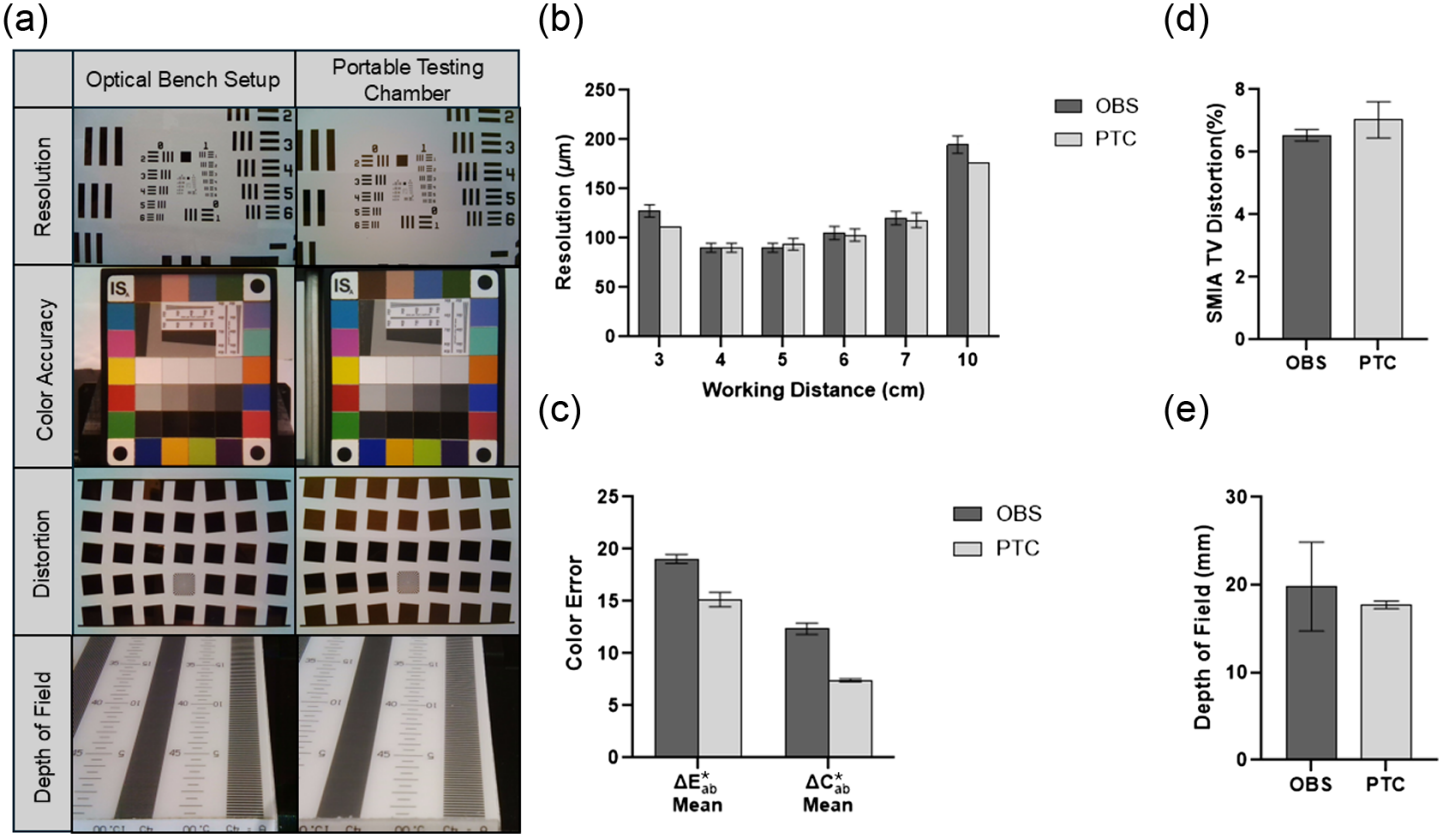
Image quality testing in the OBS vs the PTC. (a) Representative images of the glass USAF 1951 resolution target, glass RezChecker target, glass SFRplus target, and the DOF 5-15 target taken by the KeyScope in the OBS and PTC. (b) The limit of resolution was quantified in ImageJ and was comparable between the OBS and PTC across working distances of 4 to 7 cm. A lower limit of resolution was observed for the PTC at a working distance of 3 and 10 cm. (c) The percent color error was quantified using Imatest software. The $\Delta {E^{*}}_{\mathrm{ab}}$ and $\Delta {C^{*}}_{\mathrm{ab}}$ values were lower in the PTC. (d) The % SMIA TV distortion was assessed using Imatest software. There was a minimal difference between the distortion measured in the OBS versus the PTC. (E) DOF was measured in ImageJ. There was a minimal difference in DOF measured in the OBS and PTC. n=6 for all groups. Errors bars indicate standard deviation.

To validate the PTC, images of the resolution, color accuracy, distortion, and DOF targets were acquired with the KeyScope in both the PTC and OBS to enable side-by-side comparison [Fig. 6(a)]. The limit of resolution measured from the resolution target was comparable between the PTC and OBS at all working distances except for 3 and 10 cm, where the PTC yielded improved resolution [Fig. 6(b)]. In addition, the PTC yielded lower color errors [Fig. 6(c)]. Minimal differences were observed between the PTC and OBS for distortion and DOF values [Figs. 6(d)–6(e)]. All image quality metrics measured in the PTC were either similar or showed slight improvements compared with the OBS.

### 3.2 Validation of Low-Cost Test Targets

**Fig. 7:**
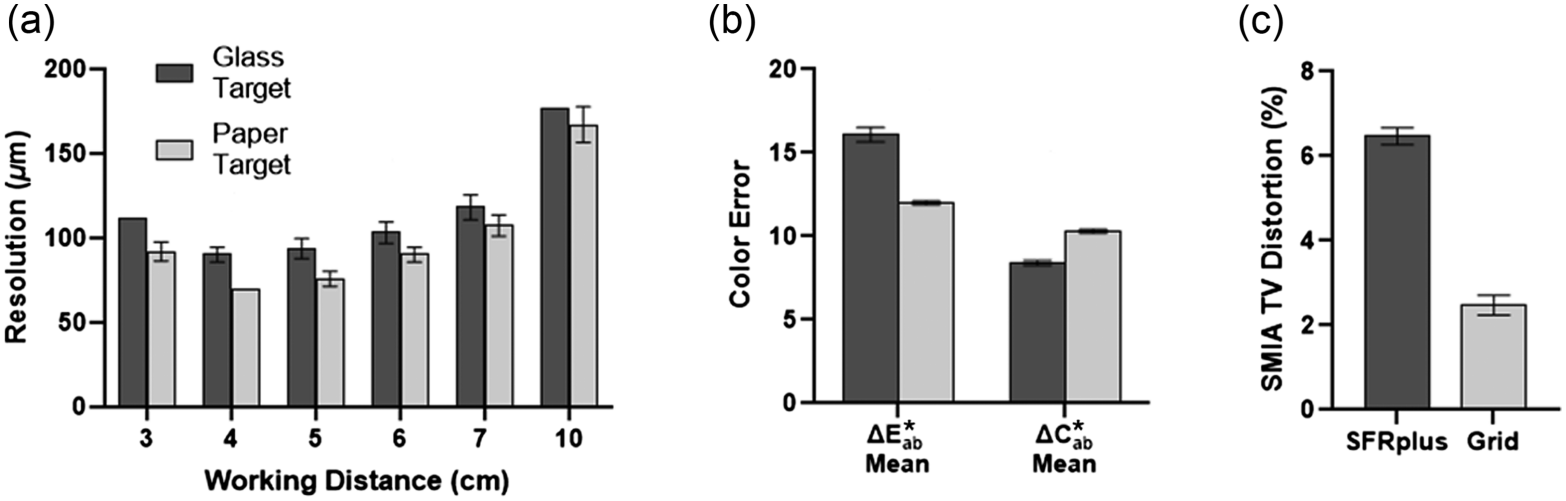
Image quality testing with glass targets versus paper targets in the PTC. (a) Resolution was measured through images of the glass and laminated paper USAF 1951 resolution target at working distances of 3 to 10 cm. The paper target displayed a slightly lower resolution at each working distance. (b) Color accuracy was assessed in the PTC using the RezChecker and paper Color Checker target. The percent error of $\Delta {C^{*}}_{\mathrm{ab}}$ and $\Delta {E^{*}}_{\mathrm{ab}}$ was quantified using Imatest software. The $\Delta {E^{*}}_{\mathrm{ab}}$ and $\Delta {C^{*}}_{\mathrm{ab}}$ values measured with the paper target were lower than the glass target. (c) Barrel distortion was assessed using the glass SFRplus target and the paper grid target in the PTC. The % SMIA TV distortion was assessed using Imatest software for both targets. The grid target resulted in lower distortion values than the SFRplus target. n=6 for all groups. Error bars indicate standard deviation.

To further improve the accessibility of our PTC design, we sought to replace the expensive and fragile glass targets with low-cost, durable paper or plastic alternatives. These low-cost targets were validated by comparing results to the standard glass targets (Fig. 7). The laminated paper USAF 1951 resolution target exhibited lower resolution at all working distances, but followed a similar pattern throughout each working distance as the glass target [Fig. 7(a)]. The color accuracy target displayed a slightly lower $\Delta {E^{*}}_{\mathrm{ab}}$ and a marginally higher $\Delta {C^{*}}_{\mathrm{ab}}$ for the paper target compared to the glass target [Fig. 7(b)]. The plastic grid target displayed lower distortion than the glass SFRplus target [Fig. 7(c)]. Finally, repeatable image quality results were obtained from representative paper targets over a year-long period (Fig. S6 in the Supplementary Material), suggesting paper target alternatives are durable.

### 3.3 Validation of Semi-Automated Image Analysis Code

**Fig. 8:**
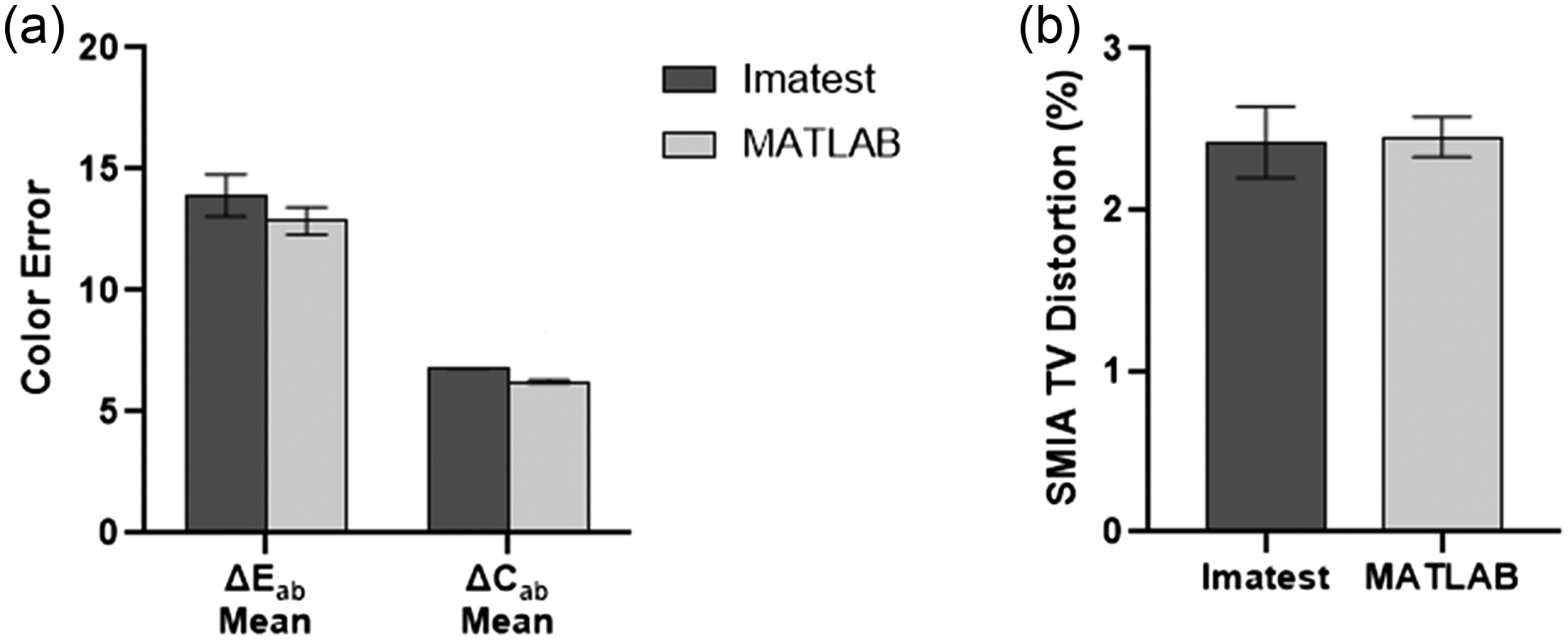
Imatest software versus semi-automated MATLAB app for measuring distortion and color accuracy. (a) Color accuracy data from Imatest and the MATLAB app is similar for $\Delta {E^{*}}_{\mathrm{ab}}$ and $\Delta {C^{*}}_{\mathrm{ab}}$. (b) Distortion analysis from Imatest and the MATLAB app was comparable. Error bars indicate the standard deviation of 5 total users who each analyzed n=4 images.

Five users assessed our distortion and color error MATLAB apps to verify that they produced comparable values to Imatest software. For the distortion analysis, the average of all 20 trials (five users, n=4 images each) was 2.42±0.19% for the Imatest analysis and 2.45±0.21% for the MATLAB app [Fig. 8(a)]. In addition, the MATLAB code resulted in similar color error values to the Imatest analysis [Fig. 8(b)].

**Fig. 9:**
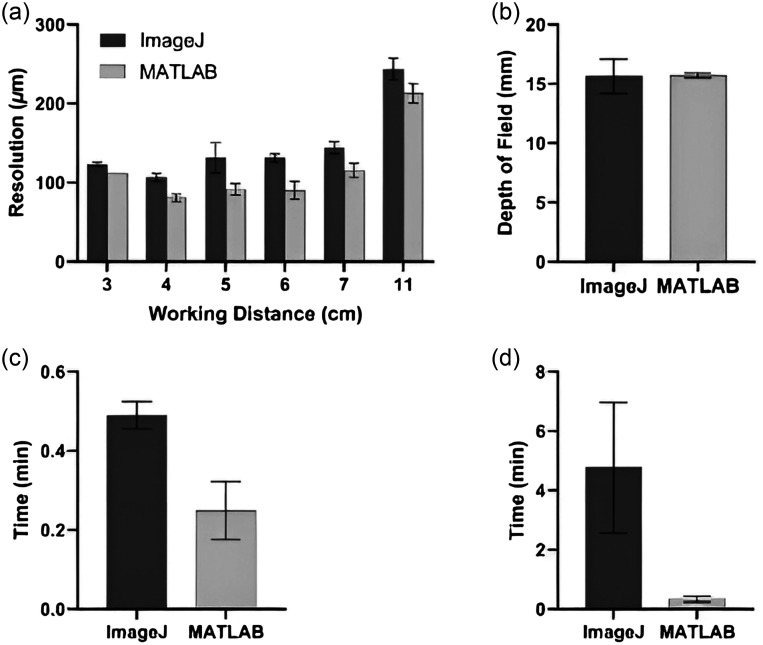
Manual analysis of the DOF and resolution with ImageJ versus semi-automated MATLAB app. (a) Resolution measured with manual ImageJ method versus MATLAB app. (b) DOF measured with manual ImageJ method versus MATLAB app comparison. (c) Average time for manual resolution analysis using ImageJ versus the MATLAB app. (d) Average time for manual DOF analysis using ImageJ versus the MATLAB app. App increased efficiency. Error bars indicate the standard deviation of five total users who each analyzed n=4 images.

Five users also tested our resolution and DOF MATLAB apps to compare with values calculated via manual ImageJ analysis methods, which is time-consuming and subject to user error. For the DOF analysis, the average of all 20 trials (five users, n=4 images each) was 15.65±1.46  mm for the manual ImageJ analysis and 14.79±0.68  mm for the MATLAB app [Fig. 9(b)]. Similarly, the resolution analysis between ImageJ and our MATLAB app was comparable across all working distances [Fig. 9(a)]. The time required for manual ImageJ versus our MATLAB app analysis was also measured for both DOF and resolution. The average time for manual analysis for DOF was 4 min 45 s compared with 11 s for the semi-automated app, which is a 96% time reduction [Fig. 9(d)].

Similarly, a 50% reduction in resolution analysis time was observed when using the MATLAB app compared to manual ImageJ analysis [Fig. 9(c)].

## 4 Discussion

To enable the testing of our low-cost laparoscope, we designed a PTC suitable for LMIC environments. The process of camera quality testing is typically very costly, starting with an OBS, consisting of an optical table, target holder components, and lighting. Glass image quality targets are expensive and image quality analysis software is costly and time-consuming. Here, we designed this process to be more simplistic, cost-efficient, and space-efficient by developing the PTC, validating low-cost targets, and developing automated MATLAB apps.

Key requirements were carefully considered in the design of the PTC, including optical target orientation and lighting.^20^ For target positioning, the target should be centered in the camera’s field of view, and the target should be oriented such that the horizontal and vertical edges are parallel to the edge of the camera frame line. The PTC target and KeyScope holders were designed to accommodate simple user adjustments to ensure the proper alignment of the optical targets relative to the KeyScope (Fig. 1). In terms of lighting, we selected the LED position that achieved the most uniform lighting and most closely matched the lux values measured in the OBS (∼600  lux). The lighting requirement specified by ISO12233-2024 is to achieve a <10% difference in brightness from the center to the edge of the target.^20^ With the placement of our LEDs on the top front, any target position within the PTC resulted in lux values with <10% deviation (Fig. 2), indicating that any illumination deviation is within regulatory standards. In addition to achieving internal lighting uniformity, the design of the PTC blocks out ambient lighting, which reduces variability. These parameters allow for a testing setup that achieves consistent lighting across different users and environments.

To validate the PTC, measurement of four image quality metrics (resolution, color accuracy, distortion, and DOF) were acquired with the KeyScope in the OBS and PTC and then compared. Overall, the PTC achieved similar or improved image quality results compared with the OBS (Fig. 6). The distortion and DOF results were comparable between the PTC and OBS, indicating similar ranges of values were obtained. In addition, the KeyScope limit of resolution results were comparable between the OBS and PTC for central working distances of 4 to 7 cm; however, the PTC displayed a lower limit of resolution at 3 and 10 cm, which is likely due to the improved lighting uniformity of the PTC. Finally, the PTC resulted in lower color errors compared with the OBS, most likely due to the difference in LED color temperature, as the color temperature can create a hue on the color target, impacting the perception of each reference color space.^21^ The PTC LEDs have a temperature of 6500 K, whereas the OBS LEDs have a temperature of 4900 K, both of which comply with regulatory light temperatures for color accuracy measurement, 5700 K ± 1000 K (ISO/TS 17321-4:2022).^22^ In addition, the PTC was enclosed eliminating any impact of ambient lighting on the perception of the color target (in contrast to the OBS). Further, a color temperature of 6500 K recapitulated daylight, which is considered ideal for color reproduction accuracy.^23^ Taken together, the PTC performed comparably or better with the OBS, confirming that the necessary image quality testing for the manufacture of the KeyScope can be performed successfully in the PTC.

We then set to decrease the cost of image quality analysis targets and found that the low-cost paper targets were comparable with the standard glass targets (Fig. 7). The paper resolution target displayed a lower resolution limit for working distances of 3 and 10 cm compared with the glass resolution target. Although there are differences at each working distance, the overall trends (i.e., a decrease in the limit of resolution from 3 to 4 cm working distances and then a gradual increase in the limit of resolution from 4 to 10 cm working distances) were comparable between the paper and glass targets, suggesting the potential application of the paper resolution target for manufacturing validation. The low-cost paper color target displayed lower color error than the RezChecker target. This difference is likely due to the different materials in the two targets (plastic versus cardboard), creating differences in the images captured with the KeyScope.^24^ Similarly, the number of reference colors differed between the two targets, with 18 color reference squares and 12 white balance squares in the RezChecker and 18 color space values and 6 white balance squares for the low-cost color target. This may lead to differences in the final average color error calculated from each target.^25^ The final paper target that we assessed was the distortion target. The quantification of this metric is less established, with multiple options approved through regulation and literature, such as the SRFplus target, dot target, and grid target.^19^ Although the SFRplus and grid targets result in different SMIA TV distortion values for the KeyScope, this difference may be due to how the calculation was performed on the differing patterns. The grid target quantified through SMIA TV distortion complied with ISO 9039,^26^ which motivates the choice of target, as it creates a simple platform to design a MATLAB app to quantify the results. Each of these low-cost cardboard or paper targets replaced the standard fragile glass targets. One possible concern of exchanging glass for paper material may be the aging of these targets; however, the image quality analysis results were repeatable over the span of a year, indicating paper targets are durable (Fig. S6 in the Supplementary Material). Furthermore, due to the fragile nature of glass, glass targets will likely require replacement as well, especially if dropped or mishandled. Ultimately, comparisons of these low-cost targets to the validated glass targets provide us with information on the expected KeyScope image quality outcomes for low-cost targets, providing a standard value for comparison when assessing the performance of KeyScopes manufactured in the future.

The previous analysis of the image quality tests utilized both ImageJ and Imatest software, which both have distinct disadvantages for manufacturing in LMICs. Resolution and DOF were analyzed through ImageJ, which required manual calculations from image intensity profiles. The manual calculations were time-consuming and led to higher inter-user variability due to the tedious nature of the calculations, which is not ideal for manufacturing KeyScope units. With the creation of semi-automated MATLAB apps, both the duration of image analysis and the inter-user variability (which is reflected in the standard deviation) decreased (Fig. 8). Color accuracy and distortion tests were analyzed with Imatest, which is an expensive software that may be inaccessible in LMICs. Thus, MATLAB apps were created to analyze color accuracy and distortion to reduce the cost of image analysis. Although there were slightly lower means calculated through our MATLAB apps than the manual image analysis for DOF and resolution, this may be partially attributed to the low standard deviation produced by the apps (Figs. 8 and 9). Further, DOF and resolution analysis require an intensity plot generation; however, ImageJ and MATLAB employ different underlying codes to provide this plot, causing inherent differences in the data produced.^27^^,^^28^ In addition, the MATLAB apps were created with a user-friendly GUI that provides directions for performing the analysis, thus creating an efficient image quality analysis process.

Opportunities for future work were identified to enable simplified quality assurance testing of the KeyScope. This includes creating optical target holders that allow for simple swapping between each image quality test target. Further, our current KeyScope is designed with a 0-deg view; however, 30-deg laparoscopes are commonly used depending on the location of the surgical field. Thus, in the future, the PTC will be modified to accommodate the 30-deg configuration. Specifically, a 30-deg spout will be incorporated into the PTC to efficiently position the camera sensor parallel to the targets. In addition, future work will include developing a low-cost, 3D-printed DOF target, as there are currently none commercially available. Further, each MATLAB analysis app was only tested with target images captured with the KeyScope. To ensure complete accuracy of the results, each app will be tested with images from different cameras, such as a commercial laparoscopic camera system. Finally, MATLAB apps will be transferred to an open-source Python or a web-based app to further increase accessibility and reduce the cost of performing image analysis. This could also enable the improvement of our image analysis algorithms via machine learning approaches once a larger data set is obtained.

## 5 Conclusion

The PTC enabled reliable image quality target testing of our low-cost KeyScope laparoscope. The PTC is preferable over a standard OBS setup for our application due to the low cost, portability, and lighting uniformity for consistent image quality measurements. Low-cost image quality test targets were validated against standard glass targets to quantify KeyScope performance. Finally, to automate image quality test analysis, semi-automated MATLAB apps were created to analyze the images, reducing the need for expensive software or time-consuming analysis methods. Overall, the PTC, low-cost targets, and automated MATLAB apps will reduce the cost, time, and complexity of KeyScope image quality validation and enable manufacturing in LMICs.

## Supplementary Material



## Data Availability

The data generated in this study are available through GitHub: https://github.com/abarnes9/Image-Quality-Analysis-Apps.
