# Supplementary material for: Development of a portable testing chamber to assess imaging performance of laparoscopes in low- and middle-income countries
Source: J Biomed Opt. 2025 Jan 30;30(1):016001. doi: 10.1117/1.JBO.30.1.016001 (PMC11781219; doi:10.1117/1.JBO.30.1.016001)
Supplement: Supplementary file 1 [file JBO_030_016001_SD001.docx]

**Supplementary Material**

Semi-automated MATLAB applications (apps) were developed for resolution, DOF, color accuracy, and distortion measurements. Section 1 contains a description of the MATLAB apps that were developed to automate the manual ImageJ analysis of the resolution and depth of field targets. Section 2 contains a description of the MATLAB apps that were developed to replace the analysis of the distortion and color targets, which were previously analyzed in expensive Imatest software. Each section has subsections describing the image analysis calculations, and the development of the MATLAB apps.

**S 1 ImageJ analysis-based code**

Previous ImageJ analysis of resolution and DOF targets were laborious and time consuming. Thus, MATLAB apps were developed to enable automated and efficient image analysis of these targets.

*S 1.1 Resolution Code development*

The steps conducted during resolution testing are illustrated in **Fig. S1**. The process begins with capturing an image of the USAF 1951 resolution target at specified working distances. To manually calculate resolution via ImageJ, the resolution target image is opened, and the user draws a vertical line through a horizontal line pair element that appears to be near the limit of resolution (i.e. where the lines are starting to blur together). The intensity profile is plotted through ImageJ, and the user finds the smallest peak and the highest trough. If the intensity value of the peak is less than double the value of the trough, then that element number is not resolvable. The user must repeat this analysis until they find the element number that passes this calculation, and that element will provide the limit of resolution, based on the established values that each element corresponds to.

To translate this process to our MATLAB app, we created a code that automatically replicates the above calculations with MATLAB functions. Through the app platform (**Fig. 5**), the user uploads the target image, which is presented to the user in greyscale. Then, the user draws a line through an entire group of preferred elements with the Draw and Process MATLAB function. This is in contrast to the manual ImageJ analysis, in which it is not recommended for the user to create the intensity profile of multiple elements simultaneously because it can be difficult to keep track of which maxima and minima belong to which line pair element.

Using the indices of the line that has been drawn on the image, the code extracts the intensity values along the line and automatically plots it. Using the find peaks function, the local maxima and minima are identified and stored in an array. The maxima and minima are then sequentially grouped into groups of three since each element contains three black lines (corresponding to minima) and two white spaces between each line (translating to maxima). The third white space maximum is not accounted for in the following calculations, because it is not within the line-pair elements. The code then performs a check using each group of maxima and minima to determine if the lowest maximum is twice that of the highest minimum. Once this is calculated for each element, the code assigns each group of maxima and minima a ‘fail’ or ‘pass’, and determines the last passed element. The user then inputs what the element at the start of the intensity plot is, and based on this input, the code determines which element was the final passing element. Then the app displays the limit of resolution based on established value that element corresponds to. If there is no passing or failing elements, the user will be prompted to retry the measurement.


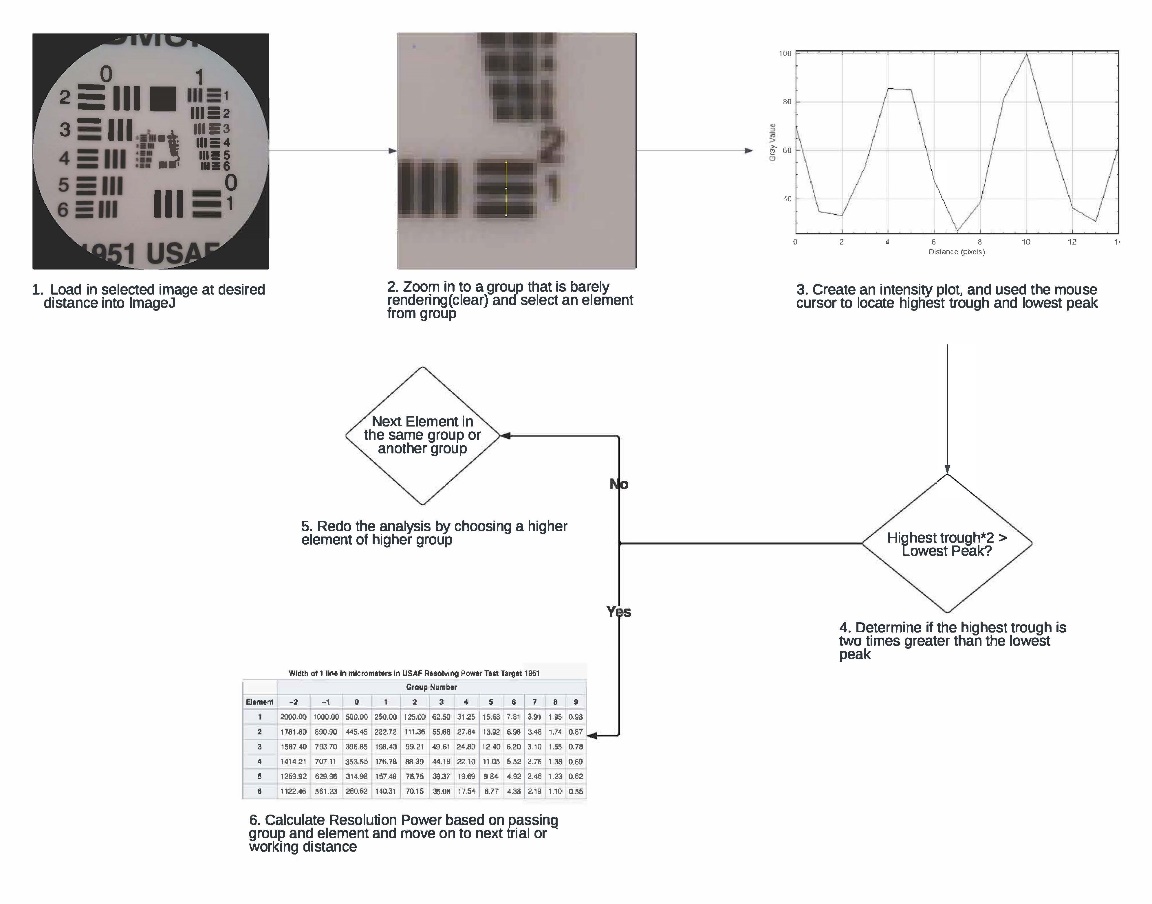


**Fig. S1** Steps conducted during resolution testing. This manual procedure uses ImageJ software to draw a line over specific line pair elements in the USAF 1951 resolution chart in step 2 and plot the intensity profile of the element in step 3. The user then determines if the element is passing in steps 4 and 5. This entire process is simplified in the MATLAB code, where the user draws a line through an entire group, and the code proceeds through step 4 and 5 automatically and presents the passing or failing elements with the determined limit of resolution.

*S 1.2 DOF Code development*

The process of the DOF manual analysis is outlined in **Fig. S2.** An image of the right column of the Edmund optics DOF target is uploaded to ImageJ, and the user draws a line through the center of the line pair column, ensuring the line stays centered and parallel to the boarders of the column. The intensity profile is plotted with ImageJ, and the difference between the first maximum (peak) and minimum (trough) is determined by the user. The user divides this value by two, which serves as the comparison threshold. The user then checks each of the following peaks to determine if the peak to trough falls below the comparison threshold value. The first peak to trough difference that is lower than the comparison threshold value is determined by the user, who then counts the number of passing peaks prior to the failure peak. The user then divides the peak count by 5, which provides the DOF in mm since there are 5 line pairs per mm.


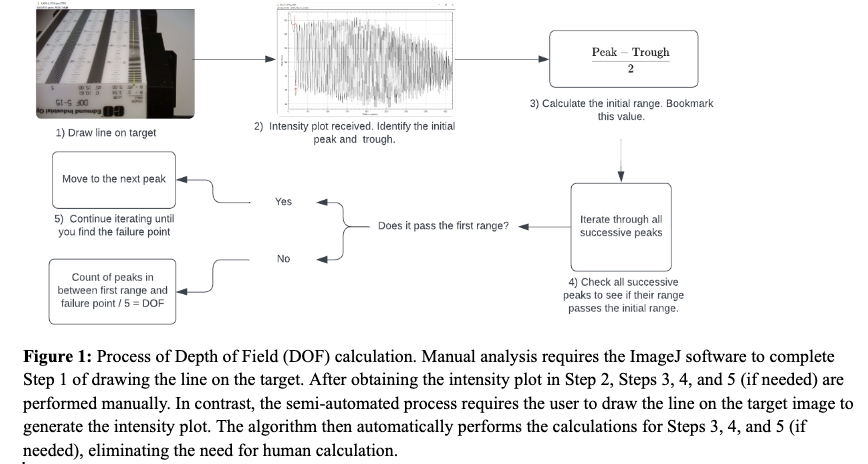


**Fig. S2** Process of the DOF calculation. Manual analysis requires the ImageJ software to complete Step 1 of drawing the line on the target. After obtaining the intensity plot in Step 2, Steps 3, 4, and 5 (if needed) are performed manually. In contrast, the semi-automated process requires the user to draw the line on the target image to generate the intensity plot. The algorithm then automatically performs the calculations for Steps 3, 4, and 5 (if needed), eliminating the need for human calculation.

This intensive manual analysis was translated into a semi-automated MATLAB app (**Fig. S3**). The user loads the image into the MATLAB app and draws a line through the right-hand column of the DOF target (similar to ImageJ). Opposed to the manual analysis, the code automatically determines the indices of line within the image, applies the information to extract the intensity values along the line, and subsequently plots the intensity profile. The findpeaks function identifies all local maxima and minima, which are stored in separate arrays. The code then calculates the initial range based on the first maximum and minimum, divides it by two, and assigns it as the DOF threshold. The code compares each minimum to maximum difference to the DOF threshold until it finds the difference that falls below the threshold. The code counts the number of maximum prior, divides the value by 5 and presents it to the user as the DOF in mm.


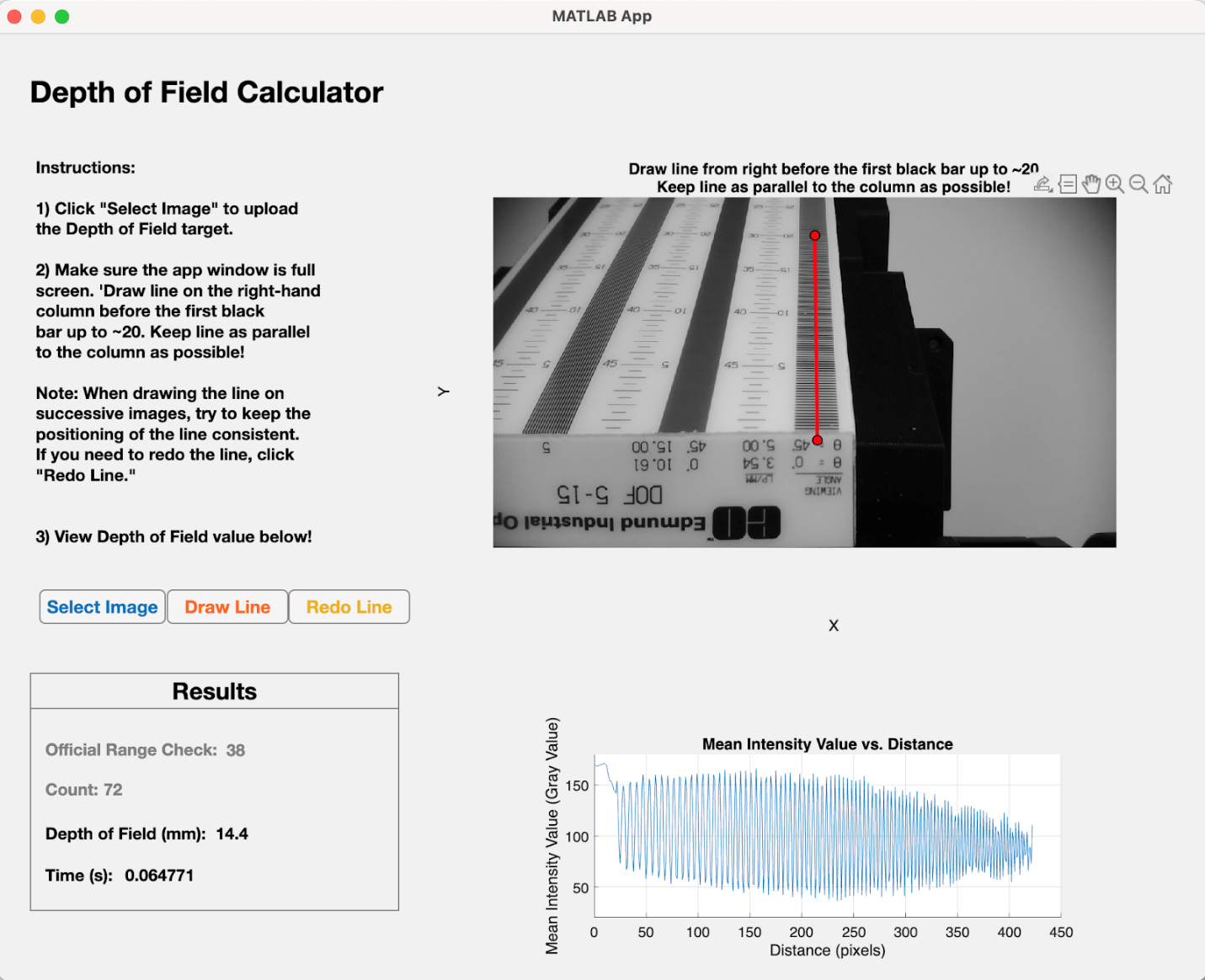


**Fig. S3** Semi-automated MATLAB DOF App user interface.

**S 2 Imatest analysis-based code**

Previous analysis of distortion and color targets were conducted in expensive Imatest software. Thus, MATLAB apps were developed to replace Imatest image analysis of these targets.

*S 2.1 Color accuracy analysis code development*

The underlying color analysis code was designed based on Imatest’s analysis methods, which calculates color error utilizing Commission on Illumination L* a* b* (CIELAB) color space values. In this system, L* represents the perceivable brightness of an image, while a* and b* represent the red/green and yellow/blue hues, respectively. The CIELAB Euclidean distance 1976 equation compares the assigned color accuracy target CIELAB reference color space values of each color square with the measured color space values from the image of the color accuracy target. Our app allows the user to align square regions of interest (ROIs) with each separate reference color squares (**Fig. S4**). The code then extracts the color information in each ROI and assigns it to a separately defined matrix. The default color space in MATLAB is RGB, so the color values from each ROI are first converted to CIELAB using built in MATLAB functions. Once converted, the L*, a*, and b* values throughout the entirety of each ROI are averaged to obtain a single measurement for each variable in each ROI. The app then applies the Euclidean distance equations for both ΔE*_ab_ and ΔC*_ab_ (equation (1) and equation (2) in the main text) for each ROI and presents the mean and maximum color differences to the user.


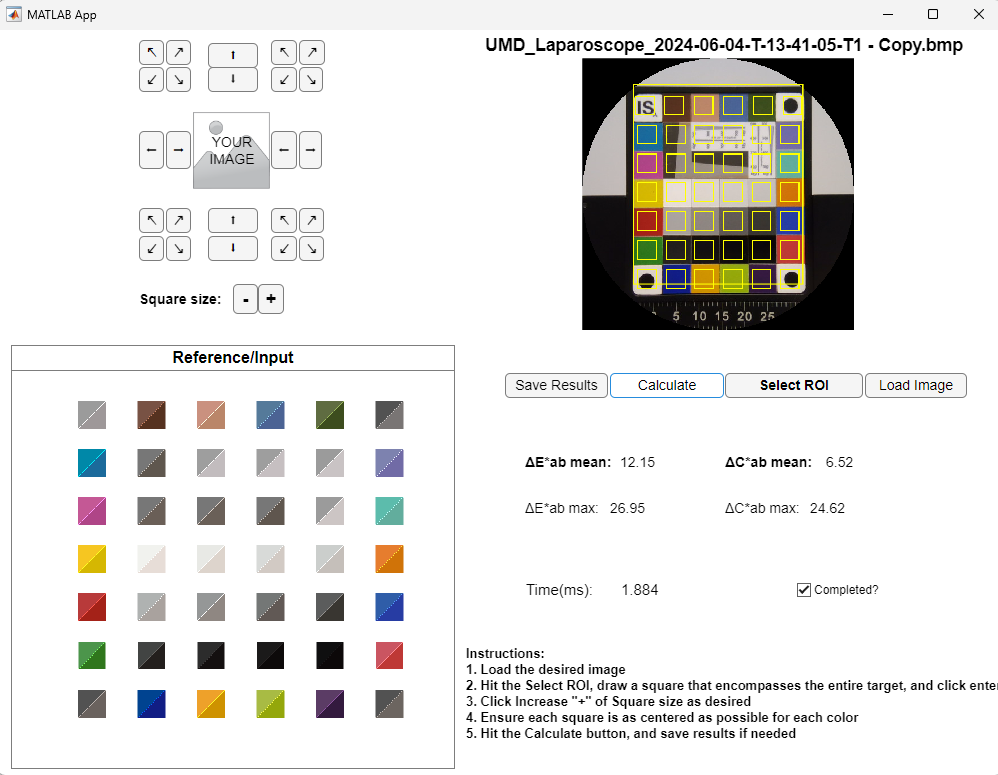


**Fig. S4** Semi-automated MATLAB App user interface when conducting color analysis of the RezChecker.

*S 2.2 Distortion analysis code development*

A MATLAB distortion app was also developed (**Fig. S5**) in place of Imatest, which calculates TV SMIA distortion. As displayed in equation (3) and (4), as well as **Fig. 3** in the main text, SMIA TV distortion is a relatively simple calculation, which averages the length of the left and right sides of the images, subtracts the length of the center from this value, and divides this by the center length to provide the proportional comparison of the center to the sides.

In our MATLAB app, the user first opens an image of a grid target. The image is converted to grayscale, and atmospheric haze is reduced with imreducehaze. The image is then made binary by assigning a threshold intensity value, determined by identifying the max intensity and subtracting 100, and any pixel intensity value below the threshold is assigned an intensity 0, while any pixel above is the threshold is assigned as 256. The image further process by skeletonizing and cleaning the skeleton, which simplifies the grid lines to be a single line of pixels across the image. The user is then shown the skeletonized image and asked to pick 6 points, four of which correspond to the left-most and right-most grid corners on the top and bottom grid lines of the target, and the final two corresponding to the center point of the top and bottom lines. The code then corrects the user input by finding the closes point on the grid lines (in the case that the user did not select directly on top of the line) and assigns the indices of the grid lines to stored variables. The distance between the top and bottom lines on the left, right, and center are calculated based on the number of pixels between the selected corner and center points. The TV SMIA equations are then used to provide the user with distortion values.


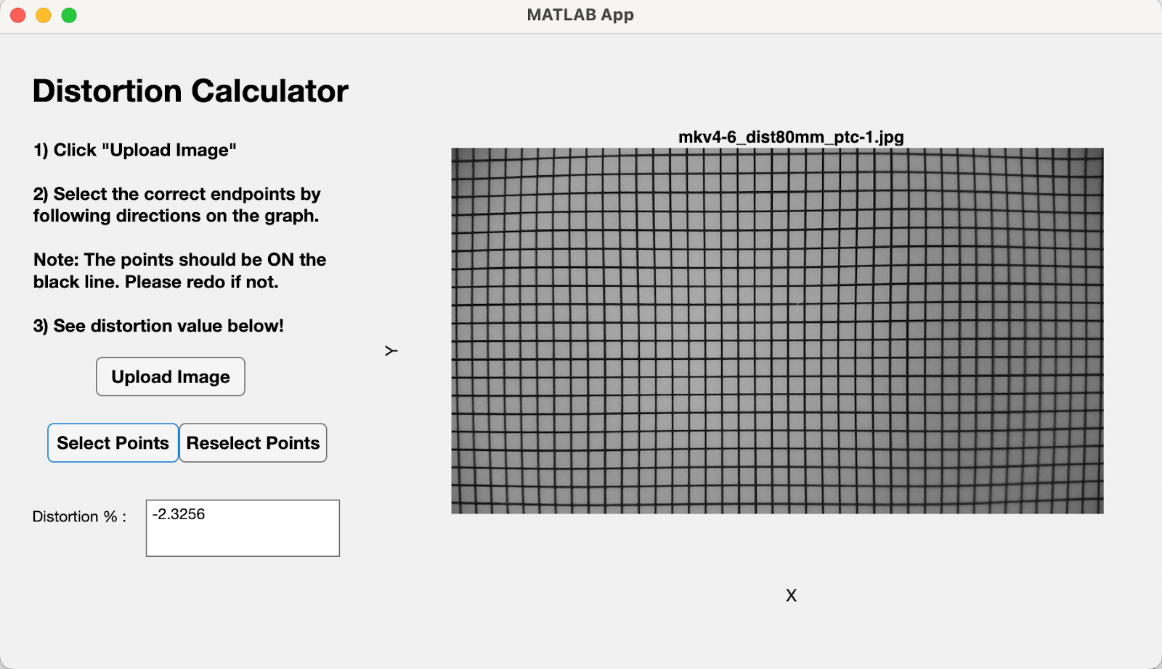


**Fig. S5** Semi-automated MATLAB Distortion App user interface.

**S 3 Paper resolution target aging**

A possible concern of using paper targets may be the aging of the target and the need for frequent replacements. However, we have found that continual use of the paper resolution target over a year did not result in deterioration or alteration of resolution measurements (**Fig. S6**).


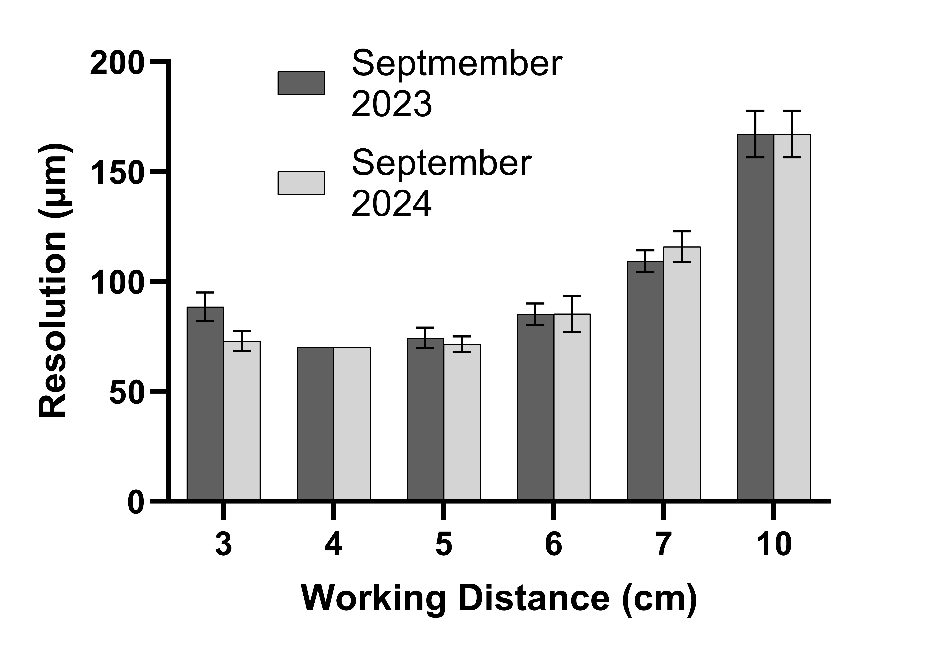


**Fig. S6** Resolution measurements in September 2023 and September 2024. Resolution was measured through images of the laminated paper USAF 1951 resolution target at working distances of 3-10 cm. The paper target displayed consistent resolution measurements from September 2023 to September 2024.
